# Applying nanotechnology to increase the rumen protection of amino acids in dairy cows

**DOI:** 10.1038/s41598-020-63793-z

**Published:** 2020-04-22

**Authors:** João Albuquerque, Susana Casal, Ricardo Nuno Mendes de Jorge Páscoa, Ingrid Van Dorpe, António José Mira Fonseca, Ana Rita Jordão Cabrita, Ana Rute Neves, Salette Reis

**Affiliations:** 1 LAQV, REQUIMTE, Department of Chemical Sciences, FFUP, Rua Jorge Viterbo Ferreira n° 228, 4050-313 Porto, Portugal; 2 Instituto de Ciências Biomédicas Abel Salazar (ICBAS), Universidade do Porto (UP), Rua Jorge Viterbo Ferreira n° 228, 4050-313 Porto, Portugal; 3 PREMIX-Especialidades Agrícolas e Pecuárias. Lda, Parque Indústrial II – Neiva, 4935-232 Viana do Castelo, Portugal; 4 LAQV, REQUIMTE, ICBAS, UP, Rua Jorge Viterbo Ferreira n° 228, 4050-313 Porto, Portugal; 5 CQM - Centro de Química da Madeira, Universidade da Madeira, Campus da Penteada, 9020-105 Funchal, Portugal

**Keywords:** Nanobiotechnology, Nanoparticles

## Abstract

The amino acid requirements of high-production dairy cows represent a challenge to ensuring that their diet is supplied with available dietary resources, and thus supplementation with protected amino acids is necessary to increase their post-ruminal supply. Lysine is often the most limiting amino acid in corn-based diets. The present study proposes the use of lipid nanoparticles as novel rumen-bypass systems and assesses their capability to carry lysine. Solid lipid nanoparticles, nanostructured lipid carriers and multiple lipid nanoparticles were considered and their resistance in a rumen inoculum collected from fistulated cows was assessed. All nanoparticles presented diameters between 200–500 nm and surface charges lower than −30 mV. Lysine encapsulation was achieved in all nanoparticles, and its efficiency ranged from 40 to 90%. Solid lipid nanoparticles composed of arachidic or stearic acids and Tween 60 resisted ruminal digestion for up to 24 h. The nanoparticles were also proven to protect their lysine content from the ruminal microbiota. Based on our findings, the proposed nanoparticles represent promising candidates for rumen-bypass approaches and should be studied further to help improve the current technologies and overcome their limitations.

## Introduction

Lysine (Lys) is commonly considered the most limiting amino acid (AA) for milk protein synthesis, particularly in high-production dairy cows fed corn-based diets, due to its presence in reduced concentrations^1^ coupled with its naturally poor intestinal absorption^2^. The AA profile of metabolizable protein (MP) must be balanced to maximize productive responses with a minimal dietary protein requirement, thus increasing the efficiency of AA utilization for milk protein production and decreasing nitrogen excretion^3–6^. The AA profile in MP can be manipulated by combining different feed ingredients and/or by supplementing diets with rumen-protected AA. However, the reported effects of rumen-protected Lys on milk and milk protein yields have been inconsistent and variable^7–10^, with negative effects on dry matter intake also being described^11^. These conflicting results may be attributed to the different conditions used in different studies, such as the crude protein (CP) concentration, MP status of the cows, and the intestinal digestibility of the undegradable dietary protein^6,12,13^.

Some lipids, particularly non-esterified saturated fatty acids, theoretically provide viable protection against ruminal digestion, as they pass through the rumen relatively unscathed, where glycerol fermentation and unsaturated fatty acid hydrogenation mainly occur^14,15^. These processes metabolize triglycerides, phospholipids or even unsaturated fatty acids, but should not damage saturated fatty acids^14,15^. Therefore, attempts to protect AA with fat coatings have been considered. However, these approaches have produced inconsistent results^5,16,17^ and their efficacy appears limited, mainly due to the poor ruminal protection that is potentially further decreased by mechanical processes such as mastication^18^. Similar approaches relying on pH resistant polymers^19^ have also been considered, but appear to suffer from similar limitations^12^. Alternative forms of Lys have also been suggested that may be resistant to ruminal digestion, such as Lys imines^20^ and hydroxymethyl-lysine^4^. However, even if these altered forms escape the rumen they might not be able to effectively release Lys, since the conditions present *in vivo* might not be ideal^21^.

Nanotechnology has emerged as a novel field with countless applications and although its use in animal nutrition is still scarce^22^, drug delivery nanocarriers have been used in medicine in numerous applications^23–26^. In particular, lipid nanotechnology has received a considerable focus due to its safe components and simple production method with easy scalability^27–31^. The application of lipid matrices as nanoparticles (NP) may help increase its efficiency through two main properties: smaller particles clear the rumen more quickly and display improved absorption at the intestinal level^32–34^.

In this study, a novel approach for rumen-bypass is proposed that relies on nanotechnology to deliver contents across the rumen. Non-esterified saturated fatty acids were chosen as the building blocks, based on their natural resistance to ruminal digestion with the benefits of a simple, cheap and scalable production method. A nanotechnological approach was chosen for its potential to increase protection from ruminal digestion. The ability of NP to resist ruminal digestion was assessed by incubating them in a fresh rumen inoculum for 24 h. The ability of the rumen-resistant NP to load Lys was also assessed for use in further studies and applications.

## Results

### Characterization of nanoparticle formulations

Three types of lipid nanoparticles were produced in this study: solid lipid nanoparticles (SLN) – NP composed of lipids that are solid at body temperature^35^, nanostructured lipid carriers (NLC) – NP composed of a mixture of lipids that are solid and liquid at body temperature^36^ and multiple lipid nanoparticles (MLN) – NP composed of a mixture of solid and liquid lipids that contain large water vacuoles^37^. NP formulations with all the proposed lipid and surfactant combinations were produced, except when using poly(vinyl) alcohol, where the formulations solidified and were thus discarded. Table 1 presents the mean diameter, polydispersion index (PdI) and zeta potential values obtained for all formulations tested.

**Table 1:** Mean diameter, PdI and zeta potential values for all tested NP, n = 3.

NP formulation	Mean diameter (nm)	PdI	Zeta potential (mV)
SLN – Stearic acid *	444 ± 11^a^	0.25 ± 0.02^a^	−35 ± 2^a^
SLN – Stearic acid #	265 ± 82^b^	0.14 ± 0.02^a^	−31 ± 1^a^
SLN – Arachidic acid *	385 ± 37^a^	0.20 ± 0.02^a^	−37 ± 1^a^
SLN – Arachidic acid #	131 ± 79^b^	0.12 ± 0.03^a^	−34 ± 1^a^
SLN – Precirol ATO 5 *	511 ± 50^a^	0.25 ± 0.01^a^	−35 ± 2^a^
SLN – Compritol 888 ATO *	556 ± 10^a^	0.17 ± 0.01^a^	−36 ± 2^a^
NLC – Stearic acid *	332 ± 40^a^	0.17 ± 0.02^a^	−39 ± 1^a^
NLC – Arachidic acid *	412 ± 25^a^	0.22 ± 0.02^a^	−46 ± 4^a^
NLC – Precirol ATO 5 *	270 ± 60^b^	0.18 ± 0.01^a^	−39 ± 1^a^
NLC – Compritol 888 ATO *	287 ± 45^b^	0.19 ± 0.03^a^	−38 ± 1^a^
MLN – Stearic acid *	216 ± 16^b^	0.41 ± 0.01^b^	−60 ± 1^b^
MLN – Arachidic acid *	208 ± 16^b^	0.39 ± 0.01^b^	−59 ± 1^b^

*Prepared with Tween 60 as the surfactant.

^#^Prepared with Tween 80 as the surfactant.

Values followed by different letters are statistically significantly different (p < 0.05).

### Ruminal resistance assay

**Figure 1:**
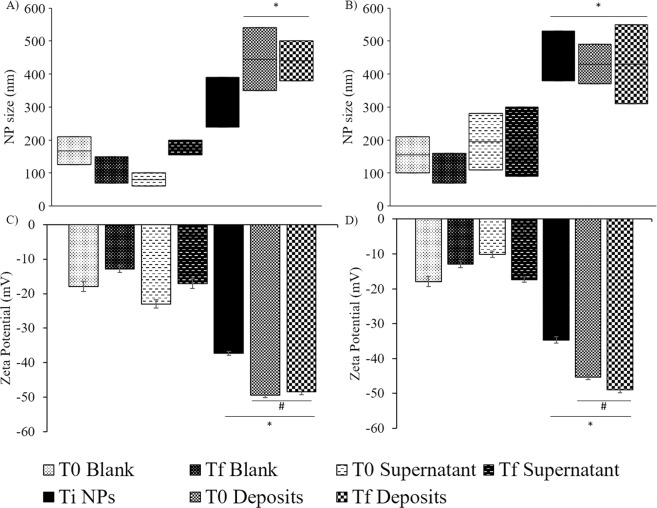
Diameter of SLN composed of stearic (**A**) and arachidic (**B**) acids with Tween 60 and zeta potential values for the same SLN (C and D, respectively) during the rumen stability assay. Results are shown for blanks and supernatants that contain bacteria (T0 and Tf) and deposits that contain NP (Ti, T0 and Tf), n = 6. *Statistically significant difference (p ≤ 0.05) from the blanks. ^#^NP were statistically significantly different (p ≤ 0.05) from Ti NP.

**Figure 2:**
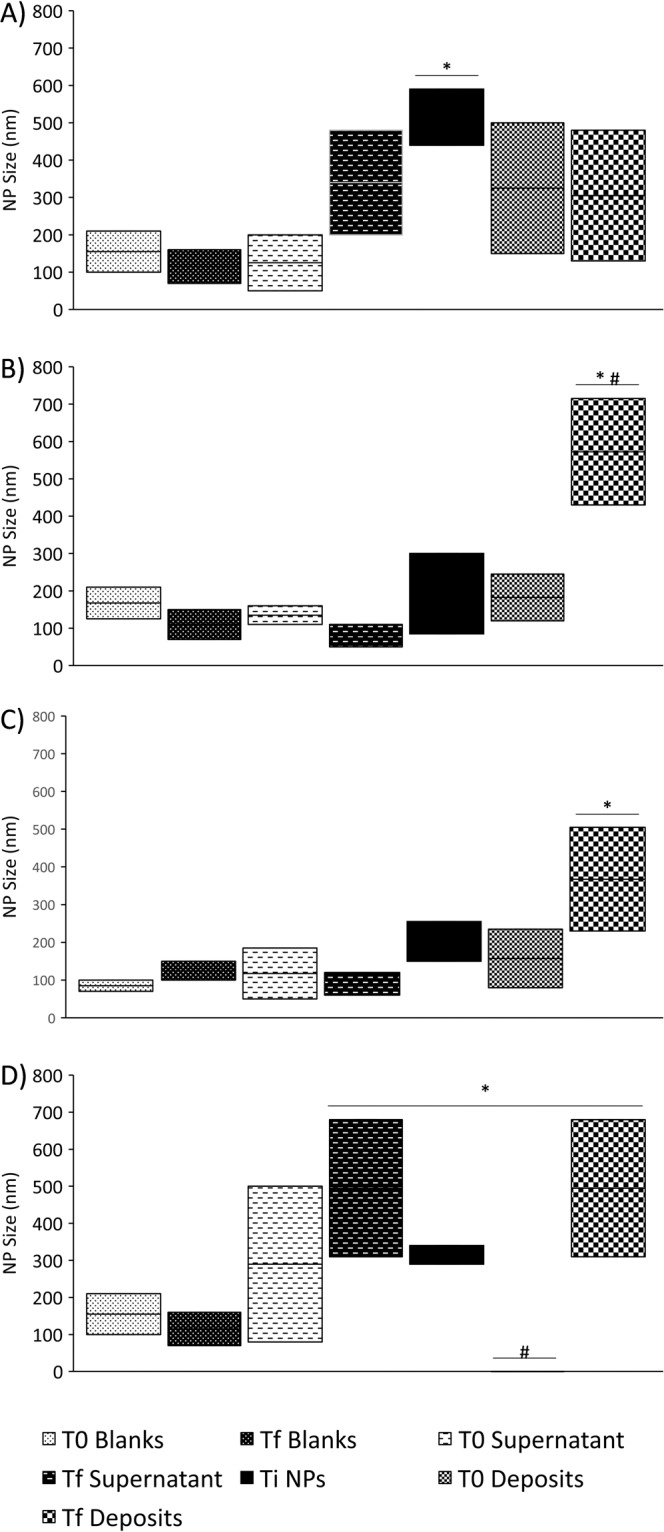
Diameters of SLN composed of arachidic acid (**A**) with Tween 80, NLC (**B**) and MLN (**C**) composed of arachidic acid measured during the rumen stability assay. Results are shown for blanks and supernatants that contain bacteria (T0 and Tf) and deposits that contain NP (Ti, T0 and Tf), n=6. *Statistically significantly different (p ≤ 0.05) from the blanks. ^#^NP were statistically significantly different (p ≤ 0.05) from Ti NP.

NP formulations were evaluated after an incubation with the rumen inoculum for 0 h (T0 samples) and 24 h (Tf samples) and were compared to the initial NP (Ti). In the ruminal stability assay of SLN composed of arachidic or stearic acids with Tween 60 (Fig. 1), supernatants presented mean diameters of approximately 100–300 nm, similar to the blanks, while deposits had diameters of 300–500 nm, indicating that the microorganisms were separated from the NP, as the latter were found in the deposit. A rumen inoculum lacking NP served as the control for both NP separation and bacterial sizes. The diameters recorded in the blanks represented bacteria and not NP, since NP were not added to these samples. Additionally, both T0 and Tf deposits presented similar sizes to the original formulations (Ti), suggesting that the NP resisted ruminal digestion. All other NP showed significant differences between the initial values and the values obtained after an incubation in the rumen inoculum. NP separation was also not possible for some formulations (Fig. 2), as the diameters of arachidic acid SLN with Tween 80 increased from 450–600 nm to 500–100 nm, while the diameters of arachidic acid NLC increased from 100–300 nm to 400–700 nm.

**Figure 3:**
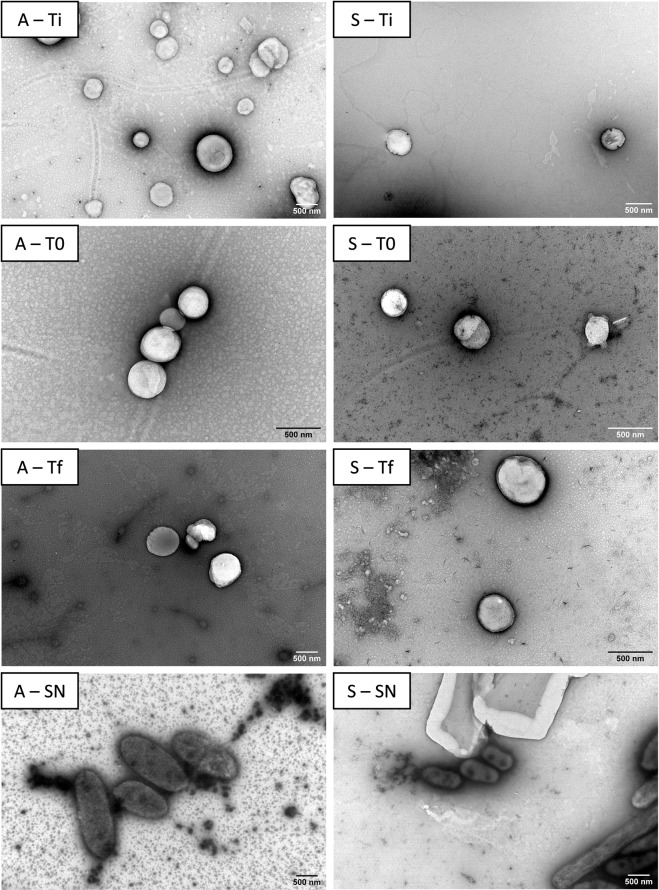
TEM photographs of both arachidic acid (**A**) and stearic acid (S) SLN with Tween 60. Ti – NP after synthesis, T0 – NP after contact with the rumen inoculum, Tf – NP after a 24 h incubation with the rumen inoculum, and SN - supernatant.

Transmission electron microscopy (TEM) photographs were captured to confirm the resistance of NP to ruminal digestion and to assess their morphology before and after an incubation with the rumen inoculum. Figure 3 presents TEM images of SLN composed of stearic or arachidic acids with Tween 60, the only NP that were able to resist the ruminal digestion, according to the previous dynamic light scattering (DLS) results. Both original NP (Ti samples) presented a spherical morphology with sizes similar to those observed with DLS (ranging from 300 to 500 nm). After 0 h (T0 samples) and 24 h (Tf samples) of incubation with the rumen inoculum, NP maintained both their size and spherical shape.

### Lysine encapsulation efficiency

The capability to encapsulate Lys inside NP was determined for SLN, NLC and MLN. The results are shown in Table 2 and refer to formulations prepared with arachidic or stearic acids along with Tween 60.

**Table 2:** Lysine encapsulation efficiency (%Lys) for SLN, NLC and MLN composed of arachidic or stearic acid with Tween 60 as the surfactant, and the amount of Lys (mg) per gram of NP, n = 3.

NP Formulations	%Lys	Lys mg/g of NP
SLN – Stearic acid	39 ± 5^a^	16 ± 2
SLN – Arachidic acid	38 ± 6^a^	16 ± 3
NLC – Stearic acid	64 ± 3^b^	27 ± 1
NLC – Arachidic acid	61 ± 5^b^	25 ± 2
MLN – Stearic acid	82 ± 3^c^	39 ± 1
MLN – Arachidic acid	92 ± 2^c^	43 ± 1

Values followed by different letters are statistically significantly different (p < 0.05).

### Evaluation of the degree of lysine protection

**Figure 4:**
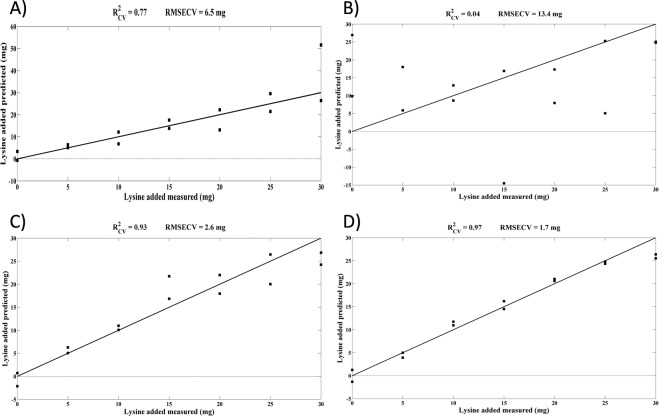
PLS models obtained at T0 (0 h of incubation) for NP with Lys added after production (**A**) or encapsulated during production (**C**) and Tf (24 h of incubation) for NP with Lys added after production (**B**) and encapsulated during production (**D**). Values of R^2^_CV_ and RMSECV are shown for each model.

The Lys encapsulation efficiency in the NP after an incubation in the rumen inoculum was evaluated by predicting the amount of Lys present in the rumen inoculum from the respective NIR spectra. Several partial least squares (PLS) models were developed, with the best PLS models obtained after pre-processing the spectra with a Savitzky-Golay filter (15-point filter width, second polynomial order and second derivative) followed by standard normal variate (SNV) and using 5 latent variables (LV). The PLS models obtained for the prediction of Lys with and without encapsulation in the NP before the incubation with the rumen inoculum (Fig. 4A,C) revealed a coefficient of determination of cross-validation (R^2^_CV_) greater than 0.75 and root mean square error of cross-validation (RMSECV) less than 6.6 mg of Lys (after eliminating the sample that predicted a very high value of Lysine in Fig. 4, the obtained R^2^_CV_ and RMSECV would be much higher and lower, respectively), indicating that the amount of Lys present in the rumen inoculum is able to be predicted. Different results were obtained from the PLS models used to predict the amount of Lys with and without encapsulation in the NP after 24 h of incubation with the rumen inoculum (Fig. 4B,D). In the former case, the R^2^_CV_ and RMSECV were 0.93 and 2.6 mg, respectively, while in the latter case, the R^2^_CV_ and RMSECV were 0.04 and 13.4 mg, respectively. Based on these results, the determination of the amount of Lys present in the rumen inoculum from the NIR spectra is feasible and Lys is efficiently encapsulated in the NP.

## Discussion

The formulations provided NP (Table 1) with sizes ranging from 130 nm to 560 nm and highly negative zeta potential values (greater than −30 mV). Significant differences in the NP size were not observed among all SLN produced with Tween 60 and between SLN formulations and the NLC produced with stearic or arachidic acid. Only the NLC produced with Precirol ATO 5 or Compritol 888 ATO were significantly smaller than the other NLC formulations and all the SLN formulations. However, MLN were similar in size to the Precirol ATO 5 or Compritol 888 ATO NLC and significantly different from all other NP produced. For SLN composed of stearic or arachidic acids, the use of Tween 80 generated NP with smaller sizes than the NP prepared using Tween 60, and these results are consistent with the literature^38^. All formulations showed PdI values ranging from 0.12 to 0.25, which indicate monodispersed NP size populations, except for MLN that possessed higher polydispersity values (approximately 0.4) indicating a wider range of NP sizes^39^. Regarding the zeta potential, all formulations showed highly negative surface charges ranging from −30 to −40 mV. These negative values were expected since both the lipids and surfactants used here have negative charges. MLN presented an even lower potential charge (−60 mV), which was the only significant difference observed. This difference in zeta potential values is probably due to the presence of large amounts of SPAN 80, which usually presents a lower zeta potential than Tween^40^. These high values for the modulus imply that NP will be less likely to aggregate and thus remain more stable as a dispersion^41^.

All these formulations, except for the formulations produced with poly(vinyl) alcohol, were incubated with the rumen inoculum, as described in the methods section. Only SLN composed of arachidic or stearic acids and using Tween 60 as a surfactant were able to resist ruminal digestion (Fig. 1). In fact, the results obtained with DLS in this stability assay are consistent with our expectations, since these NP are composed of unbound stearic and arachidic acids that pass the rumen virtually unscathed^15,42^. These 2 long-chain fatty acids (C18 and C20, stearic and arachidic acid, respectively) were specifically considered for this study since they are both saturated fatty acids with high melting points, as stated previously, and because stearic acid has the highest clearance rate among the usual lipids traversing the rumen^15^. In contrast, the remaining NP were composed of triglycerides that undergo some degree of digestion by the ruminal microbiota into glycerol and fatty acids^15,42^. Nonetheless, these lipids were considered because some lipases, such as the enzymes located in the human intestine, are only able to digest these lipids if they are emulsified in bile salts and do not function properly if they are in an NP form^43^. However, these lipids were not able to resist ruminal digestion.

SLN obtained with Tween 80 (Fig. 2A) were not as stable as SLN with Tween 60 (Fig. 1B). Both Tween detergents are composed of polyoxyethylene (20) sorbitan covalently bound to fatty acids. These fatty acids are stearic acid and oleic acid for Tween 60 and Tween 80, respectively. Stearic acid is a saturated fatty acid, whereas oleic acid contains one cis-double bond at the 9^th^ carbon; both fatty acids contain 18 carbons^44^. This small difference significantly alters the hydrophobic portion of the molecule, increasing its steric hindrance and resulting in a more fluid and potentially less stable organization of the NP surface in the presence of Tween 80^45^. Studies of the effects of these surfactants on the normal and essential functions of the ruminal microbiota have been performed and have not observed any negative effects^46,47^ and may even help to improve the functions of the ruminal microbiota^48^. In a preliminary *in vivo* trial with lactating dairy cows fed maize silage-based diets, the rumen volatile fatty acid profile was not affected by the dietary inclusion of NP (equivalent to 20 g of Lys/cow/day), with the exceptions of the proportions of propionic and butyric acids that tended to be higher and lower, respectively, after the intake of NP (Albuquerque, data not shown).

The inability of NLC and MLN to resist ruminal degradation (Fig. 2B,C) might be explained by the presence of liquid lipids in their lipid matrix, specifically Miglyol 812. The use of both liquid and solid lipids decreases the crystallinity of the lipid matrix and potentially increases substance entrapment while reducing expulsion due to a shift in the lipid form^36^. However, the ruminal conditions appear to destabilize the liquid-solid lipid matrix, exposing it to microbial digestion. Alternatively, the liquid lipids themselves naturally undergo more substantial degradation by the ruminal microbiota or a combination of these 2 phenomena occurs, rendering these NP unable to traverse the rumen. The effect of the solid lipid to liquid lipid ratio was not assessed in the current study, since the objective was to compare the resistance of several NP to ruminal digestion, and NLC and MLN were extensively degraded in the rumen. This ratio might alter the ability of these NP to resist ruminal digestion, but the results suggest that the amount of liquid lipid present must be very small and would not exert a significant effect on any other property for these NP to resist ruminal digestion.

Regarding the zeta potential results (Fig. 1C,D), the increases in modulus were expected since the rumen inoculum possesses a negative potential value that increases the overall negative charge of the mixture. The differences in the values of the deposits and the supernatants, with the latter showing similar values to the blanks, also helps confirm that the separation protocol used here was effective.

In summary, the results obtained with DLS showed that only SLN composed of arachidic or stearic acids with Tween 60 were able to resist ruminal digestion, additionally maintaining a high zeta potential that prevents their aggregation^49^. All other tested formulations showed clear signs of destruction or instability after 24 h of incubation with the rumen inoculum, and the NP sizes at Tf were very different than the size at the respective Ti. In some cases, the separation of the NP from the rumen inoculum was not possible (Fig. 2).

TEM photographs (Fig. 3) were captured to confirm the resistance to ruminal digestion and assess NP morphology. The results confirmed the conclusions obtained with the DLS analysis that both SLN composed of arachidic or stearic acids resisted digestion in the rumen inoculum for up to 24 h of incubation. Furthermore, no bacteria or other microorganisms were detected in the deposits and no NP were observed in the supernatant, indicating that the protocol effectively separated the NP from the ruminal microbiota. The observed NP sizes are adequate for intestinal uptake, although they would most likely enter the bloodstream by phagocytosis through intestinal macrophages^50^. Particles with sizes of approximately 500 nm exhibit an increased intestinal uptake^51^, suggesting that the proposed formulations are suitable for absorption in the intestine.

The NP that were able to resist ruminal digestion were subsequently assessed for the ability to incorporate Lys. However, the %Lys obtained for these particular NP was not optimal; thus, different NP that were not previously considered were tested to overcome this limitation. Different surfactants, NLC and MLN were used to increase the amount of loaded Lys and ruminal stability. MLN presented the highest %Lys (up to 90%), most likely due to the presence of large aqueous vacuoles within the lipid matrix and the hydrophilic nature of Lys. Additionally, as these vacuoles form prior to the NP dispersion, all Lys added is expected to be trapped in the internal vacuoles and not in the exterior aqueous phase that surrounds the MLN^37^. The NLC also displayed relatively high %Lys values of up to 65% while the SLN reached reasonable values of up to 40%. The higher values observed for the NLC than the SLN might be attributed to the presence of liquid lipids in the NP matrix that confers a less crystalline structure with more cavities and defects in which the encapsulated molecules can be trapped^52,53^. This structure may also reduce the effects of shifts in lipids (changes that occur in the lipid structure over time and lead to a more stable polymorphic structure) that may cause the encapsulated molecules to be expelled^36^. SLN are composed of only solid lipids and form a crystalline structure of hydrophobic molecules, resulting in fewer locations for molecule entrapment^53^ and thus a lower %Lys. No significant differences were observed in the %Lys between SLN composed of arachidic or stearic acids with different surfactants. However, both formulations presented a more than reasonable value, particularly considering the highly hydrophilic nature of the tested molecule. In summary, the highest %Lys value was obtained for MLN (80–90%), followed by NLC (approximately 60%) and SLN (approximately 40%). The %Lys value varied significantly among the different types of NP but remained very similar within each type of NP, despite the use of different lipids. Although higher %Lys values were observed for MLN, neither these formulations nor the NLC were able to resist ruminal digestion, as described above. A comparison between the Lys loading efficiency of the proposed NP and the already existing rumen-protected products was not possible at this time, as the data are not available or the results reported in the literature are not consistent, with different studies testing the same products and obtaining different values for the available Lys content^11,12,16^.

The PLS models developed using the samples before incubation revealed the feasibility of determining the amount of Lys with and without encapsulation in the rumen inoculum, since high R^2^_CV_ were obtained in both cases (Fig. 4A,C). The values of R^2^_CV_ and RMSECV were expected to be similar for the prediction of the amount of Lys encapsulated in the NP or without encapsulation. The results were slightly different mainly because one of the samples with the highest amount of Lys (30 mg) without encapsulation predicted a higher amount of Lys. Nonetheless, the objective of these assays was not to develop accurate and robust PLS models for predicting the amount of Lys in the rumen inoculum (more samples would be needed to validate the model) but to confirm the feasibility of determining the amount of Lys present in the rumen inoculum based on NIR spectra. Therefore, the results obtained here confirm the possibility of determining the amount of Lys present in the rumen inoculum.

The PLS models developed using the samples with and without Lys encapsulation and with an incubation time of 24 h were used to determine the efficiency of Lys encapsulation in the NP. The R^2^_CV_ and RMSECV obtained for the PLS model using encapsulated Lys after 24 h of incubation of 0.93 and 2.6 mg, respectively, were very similar to the values obtained for the same samples of Lys encapsulated before incubation. For the samples without encapsulation that were measured after 24 h of incubation, the obtained R^2^_CV_ and RMSECV, 0.04 and 13.4 mg, respectively, revealed that the amount of Lys present was not able to be predicted. This difference compared with the model established at 0 h of incubation may be associated with the chemical changes that occur in the rumen that may have contributed to the degradation/modification of Lys, most likely by the ruminal microbiota. Therefore, the results obtained after an incubation of 24 h indicate that the encapsulation of Lys in the NP is efficient or otherwise the R^2^_CV_ value would be close to 0 and the RMSECV value would be high.

## Conclusions

The goal of this study was to develop a lipid-based nanoformulation capable of bypassing the rumen without being digested and to determine whether these formulations could be loaded with Lys. Several types of lipidic NP: SLN, NLC and MLN, as well as different lipids and surfactants were considered and tested in this study, all of which were assessed for their ability to resist digestion in the rumen and their ability to be loaded with Lys. All these formulations were produced using an organic solvent-free method based on sonication that can be scaled. SLN composed of arachidic or stearic acids and Tween 60 bypassed microbial digestion in the rumen for periods of up to 24 h. Furthermore, the NP were loaded with Lys, a highly hydrophilic AA, although the %Lys encapsulated was not very high for the only NP that were able to resist digestion, thus further optimization and studies are required. Moreover, encapsulated Lys is able to resist digestion in the rumen, indicating that the proposed NP are able to protect this amino acid for up to 24 h. Prior to an *in vivo* evaluation of the NP formulation developed here, the results must be further validated by conducting *in vitro* trials with a greater number of inoculum donor animals fed different diets to evaluate both the resistance of NP and their effects on ruminal fermentation parameters. The release of Lys from the NP after leaving the rumen must be evaluated to assess its availability for absorption in the small intestine. In conclusion, the proposed formulations, specifically the SLN composed of arachidic or stearic acids with Tween 60, are capable of resisting digestion in the rumen and loading Lys, showing promise and potential for further applications in rumen-bypass delivery systems. In fact, by relying on smaller particles that are denser than the ruminal fluid, these carriers would traverse the rumen faster and result in an even greater reduction in degradation compared to conventional approaches. Further studies and tests are required, particularly to improve the %Lys, but the suggested formulations might help overcome the current limitations in the field in the future by more effectively protecting the contents from digestion in the rumen.

## Materials and Methods

### Materials

Stearic acid and SPAN 80 were purchased from Merck (Merck KGaA, Darmstadt, Germany). Arachidic acid, Tween 60, Tween 80, poly(vinyl alcohol), L-lysine monohydrochloride, lithium carbonate, dansyl chloride, methylamine hydrochloride, triethylamine and sodium acetate were purchased from Sigma-Aldrich (St. Louis, MO, USA) and Miglyol 812 was purchased from Caelo (Caesar & Loretz GmbH, Hilden, Germany). Precirol ATO 5 and Compritol 888 ATO were kindly provided by Gattefossé (Saint Priest Cedex, France). L-Phenylalanine ethyl-ester hydrochloride was purchased from Fluka (Fluka Chemie GmbH, Buchs, Switzerland), acetic acid was obtained from VWR Chemicals (VWR International S.A.S., Fontenay-sous-Bois, France) and acetonitrile and methanol were obtained from Honeywell (Honeywell Riedel-de Häen AG, Seelze, Germany). Aqueous solutions were prepared with double-deionized water (Arium Pro, Sartorius AG, Göttingen, Germany).

## Methods

### Production of nanoparticle formulations

Three types of lipid nanoparticles were produced using an organic solvent-free emulsification-sonication method: SLN^35^, NLC^36^ and MLN^37^. The proposed nanoparticles were selected for their properties. MLN were selected because their interior vacuoles would be the most suitable carriers of Lys^37^, considering their strong hydrophilic nature^54^. SLN and NLC, despite exhibiting a lower Lys loading potential, are relatively simpler to produce and thus would be easier to produce at an industrial scale^55^.

Table 3 presents the different combinations of lipids and surfactants used to prepare the nanoparticles. Lipids were chosen for their natural resistance to rumen digestion, and different lipids were selected for their high melting point (>65 °C) to enable their application in compound feeds exposed to temperatures of approximately 70 °C during production. Meanwhile, the surfactants were chosen for their low cost and because they are already used in several food products. All the tested lipid and surfactant combinations are listed in Table 3; however, they were not all tested at the same stage in this study, but were considered possible alternatives based on the results of preliminary tests. Thus, not all possible combinations were tested.

**Table 3:** Different combinations of solid lipids, liquid lipids and surfactants tested for NP production.

Type of NP	Solid lipid	Liquid lipid	Surfactant
SLN	Stearic acid	-------------	Tween 60
SLN	Stearic acid	-------------	Tween 80
SLN	Stearic acid	-------------	poly(vinyl alcohol)
SLN	Arachidic acid	-------------	Tween 60
SLN	Arachidic acid	-------------	Tween 80
SLN	Arachidic acid	-------------	poly(vinyl alcohol)
SLN	Precirol ATO 5	-------------	Tween 60
SLN	Compritol 888 ATO	-------------	Tween 60
NLC	Stearic acid	Miglyol 812	Tween 60
NLC	Arachidic acid	Miglyol 812	Tween 60
NLC	Precirol ATO 5	Miglyol 812	Tween 60
NLC	Compritol 888 ATO	Miglyol 812	Tween 60
MLN	Stearic acid	Miglyol 812	SPAN 80 + Tween 60
MLN	Arachidic acid	Miglyol 812	SPAN 80 + Tween 60

Precirol ATO 5 consists of esters of palmitic (C16) and stearic (C18) acids, and the diester fraction is predominant.

Compritol 888 ATO consists of mono-, di- and triesters of behenic acid (C22), and the diester fraction is predominant.

Regarding SLN and NLC production, lipids and surfactants were melted at 85 °C in a water bath. Five hundred milligrams of solid lipid for SLN, 350/150 mg of solid/liquid lipids for NLC and 100 mg of surfactants for both SLN and NLC were used to prepare the formulations. A 5.68 mg/mL Lys solution was prepared in ultra-pure water using L-Lys monohydrochloride and heated at the same temperature. Next, 4.4 mL of the Lys solution were added to the melted lipids and the resulting emulsion was sonicated using a probe-type sonicator (model VCX-130 with a VC 18 probe, Sonic & Materials Inc., Newtown, CT, USA). SLN were sonicated for 5 min with an 80% amplitude, and NLC were sonicated for 15 minutes with a 70% amplitude. The resulting nanoemulsions were then stored at room temperature (RT) until further use.

For the MLN, 1000 mg of solid lipid, 3000 mg of liquid lipid and 1800 mg of the surfactant SPAN 80 were blended and melted at 85 °C in a water bath. A 55 mg/mL Lys solution was heated at the same temperature as the lipids, and then 5 mL of this solution were added to the melted lipids. The resulting emulsion was then sonicated for 14 min at a 70% amplitude at 85 °C, followed by 10 min of rest at RT. A 1 mg/µL Tween 60 solution was added (650 μL) to the emulsion and again sonicated for 8 min at 60% amplitude at 85 °C. The resulting emulsion was incubated at RT to allow it to solidify, resulting in an ointment-like emulsion. This emulsion was stored in this form until further use, after which it was dispersed in water when needed to form a nanoemulsion formulation^37^.

### Characterization of nanoparticle formulations

The NP formulations were characterized in terms of the mean diameter, PdI or size distribution profile and zeta potential. The mean diameter and PdI were determined using DLS with a 90Plus Particle Size Analyzer (Brookhaven Instruments Corporation, Holtsville, NY, USA), and the zeta potential was assessed through phase analysis light scattering (PALS) using a ZetaPALS Zeta Potential Analyzer (Brookhaven Instruments Corporation, Holtsville, NY, USA). A wavelength of 660 nm, a temperature of 20 °C, and a detection angle of 90° were used to record the measurements and the refractive indexes were 1.59 and 1.33 for the NP and the solvent (water), respectively. All samples were diluted in ultra-pure water.

The formulations were subjected to a TEM analysis to assess the NP morphology and size. The NP formulations were diluted 100x in ultra-pure water. Samples were prepared by dropping 10 µL of the NP dispersion on a copper-mesh grid, and the excess was removed using filter paper. Uranyl acetate was used as the contrast agent (10 µL of a 0.75% solution were added to the grid), and the excess was removed with filter paper. The grids were observed using a JEM-1400 transmission electron microscope (JEOL Ltd., Tokyo, Japan) at an accelerating voltage of 80 kV.

### Rumen resistance assay

Ruminal contents were obtained from 2 multiparous (number of parity = 2) adult Holstein cows aged 5 and 6 years, both dry and not pregnant that were fitted with a ruminal cannula (10 cm diameter; Bar Diamond Inc., Parma, ID). The cows were housed at the Vairão Agricultural Campus of Abel Salazar Biomedical Sciences Institute, University of Porto (ICBAS-UP; Vila do Conde, Portugal). Cows were handled in strict accordance with good animal practice as defined by the national authority and European Union Directive 2010/63/EU. Experimental animal procedures were approved by the Local Animal Ethics Committee of ICBAS-UP, licensed by the Portuguese Directorate-General of Food and Veterinary Medicine of the Ministry for Agriculture and Sea, and conducted by trained scientists (FELASA category C). Each cow was fed a total mixed ration based on corn silage or haylage and had fresh drinking water available at all times. Ruminal contents were collected before the morning meal from the 4 quadrants of the rumen and placed in a 4 L pre-warmed (39 °C) thermal jug. In the laboratory, the ruminal digesta of each cow was homogenized and strained through 4 layers of linen cloth at 39 °C in O_2_-free CO_2_ atmosphere. The interval between the collection of the ruminal contents and incubation never exceeded 60 min. One part of the strained ruminal fluid, pH 6.3, was diluted anaerobically into 4 parts of Kansas State Buffer^56^ and mixed at 39 °C in an O_2_-free CO_2_ atmosphere. Thirty millilitres (25 mL of buffered ruminal fluid and 5 mL of each NP formulation) were dispensed anaerobically into 125 mL serum bottles (Sigma-Aldrich Inc., St. Louis, MO, USA) containing 250 mg (dry matter) of wheat straw, sealed with butyl rubber stoppers and aluminium crimp caps (Sigma-Aldrich Inc., St. Louis, MO, USA), and incubated in a water bath at 39 °C. Each NP formulation, as well as blanks containing no NP, were incubated in quadruplicate. Two of these replicates of each sample and blanks were placed in an ice bath immediately after the addition of the NP formulation to serve as negative controls (T0 samples). Fermentations were stopped after 24 h by cooling the bottles in an ice-slurry bath (Tf samples). The samples were subsequently compared with NP that did not contact the rumen inoculum (Ti samples).

The contents of the flasks were transferred to Falcon tubes and centrifuged at 500 g for 5 min to separate any remaining feed and the heaviest microorganisms, such as protozoa, from the NP. The supernatant was collected and centrifuged at 30000 g for 20 min to separate the NP from the bacteria and other microorganisms that did not deposit in the first centrifugation. Both supernatants and deposits were collected for characterization in terms of size, PdI, zeta potential and morphology. This procedure was also performed on the rumen inoculum and buffer mixture without the addition of any NP formulation (blanks), and these results were used as controls to verify the separation of the NP and rumen inoculum.

### Lysine encapsulation efficiency (%Lys)

The %Lys was determined by calculating the difference between the total amount of Lys added to the preparation of NP and the amount of Lys that remained outside the NP after the encapsulation process.

First, the NP suspensions were diluted 100x and filtered with Ultracell-50 kDa Amicon Ultra Centrifugal Filters (EMD Millipore, Darmstadt, Germany) at 2200 *g* for 33 min using an Allegra X-15R centrifuge (Beckman Coulter, Pasadena, CA, USA). The supernatant containing the non-encapsulated Lys outside the NP was then collected for quantification.

Because Lys is a non-chromogenic substance, derivatization with an agent that may add chromogenic or fluorescence properties to the AA is required for further quantification^57^. The chosen agent was dansyl chloride, as it is widely used to quantify AA^58,59^. Briefly, 100 µL of a dansyl chloride solution (2.6 mg/mL in acetonitrile) were added to 100 µL of sample, homogenized by vortexing, and incubated for 30 min at 60 °C in the dark. Dansylated L-lysine was quantified using high-performance liquid chromatography, and L-phenylalanine ethyl-ester was used as an internal standard (IS). The chromatographic conditions were adapted from the literature^60^. Two distinct pumps were used, both of which were Jasco PU-2080 plus intelligent HPLC pumps, and the automated injector was a Jasco AS-2057 Plus Intelligent Sampler. The column was a Kinetex EVO C18 100 Å column (100.0 × 3.0 mm, 2.6 µm). The detector was a Jasco FP-920 Intelligent fluorescence detector and the excitation and emission wavelengths were 330 nm and 508 nm, respectively. The aqueous phase (A) was composed of 0.02 M sodium acetate and 0.02% triethylamine, with the pH adjusted to 4.5 with acetic acid, while the organic phase was a 1:9 mixture of A and methanol. The flow rate was 400 µL/min and the injection volume was 20 µL. The total run time was 22 min using the following gradient: 0 min: 47% (A), 13 min: 14% (A), 15 min: 14% (A), 17 min: 47% (A), and 22 min: 47%. A calibration curve was constructed using standard solutions of L-Lys plus the IS at final concentrations of 200, 100, 50, 25, 12.5, 6.25, 1 and 0 µM. The Lys retention time was 13.3 min, while the IS peak appeared at 15.8 min.

### Recording the NIR spectra

The NIR spectra of lyophilized samples of rumen were recorded using a Fourier transform near infrared spectrometer (FTLA 2000, ABB, Québec, Canada) with an indium-gallium-arsenide (InGaAs) detector and controlled using Bomen-Grams software (version 7, ABB, Québec, Canada). Four sets of samples were considered: NP with Lys added during the synthesis, both at 0 h and at 24 h of incubation with the rumen inoculum and NP with Lys added after the synthesis, both at 0 h and at 24 h of incubation with the rumen inoculum. Each spectrum was obtained from an average of 64 scans within a wavenumber interval of 10000–4000 cm^−1^ and a resolution of 8 cm^−1^ in diffuse reflectance mode. The lyophilized samples were transferred to borosilicate flasks before spectra were recorded. For each sample, a total of three spectra were recorded and then averaged for further data analysis. Therefore, 56 spectra were acquired. The background was corrected using a reference substance (Teflon).

The curves were constructed using solutions of 0.0, 1.1, 2.2, 3.3, 4.4, 5.5 and 6.6 mg/mL Lys during production in the case of the encapsulated samples, as described in the nanoparticle formulation production section,. Alternatively, Lys was added to non-loaded NP after production to achieve similar concentrations.

### Multivariate NIR spectral data analysis

The amount of lysine was predicted by comparing the acquired near infrared spectra with the experimental values of Lys using PLS models^61^. Before the application of any chemometric tool, the spectra were mean-centred. Different pre-processing techniques, including SNV and the Savitzky-Golay filter (with different filter widths, polynomial orders and first and second derivatives), were tested individually and in combination to optimize the PLS models. The optimal number of LV was selected using a leave-one-out cross-validation procedure to prevent model over-fitting. All PLS models were developed for the whole mean-centred spectrum. The developed PLS models were evaluated by calculating the RMSECV and the R^2^_CV_. The RMSECV was calculated using the following equation:

1 $$RMSECV=\sqrt{\frac{{\sum }_{i=1}^{N}({\hat{Y}}_{i}-{Y}_{i})}{N}}$$

where *N* is the number of samples, *Y*_*i*_ is the experimental result for sample *i* and ${\hat{Y}}_{i}$ is the value obtained with the cross-validation of each sample.

All calculations were performed using MATLAB version 8.6 (MathWorks, Natick, USA) and PLS Toolbox version 8.2.1 (Eigenvector Research Inc., Wenatchee, USA).

### Statistical analysis

All statistical analyses were performed with IBM SPSS Statistics (SPSS 25.0, Armonk, NY, USA). Results are presented as the mean values ± standard deviations from a minimum of 3 independent experiments. Two-tailed Student’s t-test and one-way analysis of variance (ANOVA) were performed to compare dependent samples and multiple groups of independent samples, respectively. When the groups presented statistically significant differences (p ≤ 0.05), the differences between the respective groups were compared with a post hoc test (Tukey’s test, p ≤ 0.05).

## Data Availability

No datasets were generated or analysed during the current study.
